# Clinical drug response can be predicted using baseline gene expression levels and *in vitro* drug sensitivity in cell lines

**DOI:** 10.1186/gb-2014-15-3-r47

**Published:** 2014-03-03

**Authors:** Paul Geeleher, Nancy J Cox, R Stephanie Huang

**Affiliations:** 1 Section of Hematology/Oncology, Department of Medicine, University of Chicago, Chicago, IL 60637, USA; 2 Section of Genetic Medicine, Department of Medicine, University of Chicago, Chicago, IL 60637, USA

## Abstract

We demonstrate a method for the prediction of chemotherapeutic response in patients using only before-treatment baseline tumor gene expression data. First, we fitted models for whole-genome gene expression against drug sensitivity in a large panel of cell lines, using a method that allows every gene to influence the prediction. Following data homogenization and filtering, these models were applied to baseline expression levels from primary tumor biopsies, yielding an *in vivo* drug sensitivity prediction. We validated this approach in three independent clinical trial datasets, and obtained predictions equally good, or better than, gene signatures derived directly from clinical data.

## Background

Identifying and applying molecular biomarkers to predict response to medication is particularly important for drugs with a narrow therapeutic index, for example chemotherapeutic agents, because response is highly variable and side effects are potentially lethal [1,2]. Many studies have been conducted with this objective but only a handful of markers can reproducibly predict chemotherapeutic response in the clinic [3]. It is anticipated that the number of biomarkers discovered will rise as high-throughput sequencing becomes cheaper and more pervasive [3,4]; however, the effect size of these markers is generally small, since drug response is typically a complex trait, usually influenced by many genomic and environmental factors [3,4]. Thus, it has been hypothesized that methods that consider the cumulative effect of many markers, may predict complex phenotypes (like drug response) more accurately. Consequently, some researchers have recently developed sophisticated methods that incorporate all of the data in a genome. For example, there has been some success in using whole-genome SNP or sequence data to predict complex traits [5,6].

In cancer, genomic aberrations and aneuploidy are common, which means that it is difficult to obtain reliable SNP or genome sequences directly from tumors [7]. However, the quantification of whole-genome gene expression levels from primary tumor biopsies is straightforward and has been successfully applied for many years [8,9]. Unfortunately, prediction using gene expression microarray data has traditionally been fraught with reproducibility issues [10]. One of the major concerns is that gene expression estimates, generated on different microarray platforms or even in different batches, are not always consistent [11]. Several analytical approaches have recently been suggested to address this problem and a large-scale comparison has found that some of these methods reliably correct for these biases [12]. Also, multiple studies have compared the performance of various algorithms for predicting survival phenotypes from microarray expression data [13,14]. These have found that ridge regression (a type of regularized linear regression that can include the expression of all genes in the model) performed best, or was consistently amongst the best performing methods. However, gaps remain in the utility of these tools in predicting clinical phenotypes.

Here, we present an approach that integrates several of these recently developed computational and statistical tools to predict *in vivo* drug response, using models trained on cell line data (see Materials and methods). For model development, the approach was applied to recently released data from the Cancer Genome Project (CGP) [15], consisting of baseline (i.e. before drug treatment) gene expression microarray data and sensitivity to 138 drugs in a panel of almost 700 cell lines. Our results demonstrate that by building a statistical model from these data, it is possible to capture a significant proportion of the variability in drug response in patients. The Cancer Cell Line Encyclopedia [16] has an additional large panel of cell lines, for which it is possible to construct such models, although here, we focus on the CGP, because those cell lines have been screened against more drugs.

To test our approach, we identified clinical trial datasets that had assessed tumor gene expression before drug treatment (using expression microarrays) and had subsequently measured a clear drug response phenotype. Using these data, we can test whether our models derived from cell lines capture a significant proportion of the variability in drug response in patients. The clinical datasets must fulfill the following criteria. Firstly, the clinical trial data (both baseline tumor expression and post-treatment drug response) must be publicly available and easily accessible to allow other researchers to reproduce the results. Patients must have been treated with monotherapy, rather than a combination of drugs, as multi-drug regimes would clearly confound the results. The data must have been published and not retracted. A reasonable number of clinically evaluable samples (>20) are required for statistical power. Finally, sensitivity to the particular drug (as measured by the concentration required for 50% of cellular growth inhibition (IC_50_)), must have been quantified in the CGP cell lines, because we cannot create suitable models otherwise. To our knowledge, there are four existing datasets that fulfill these criteria [17-20] and the results of our analysis of these data are presented below. Interestingly, the four trials were for three different types of cancers treated with either cytotoxic or targeted agents.

## Results

**Figure 1:**
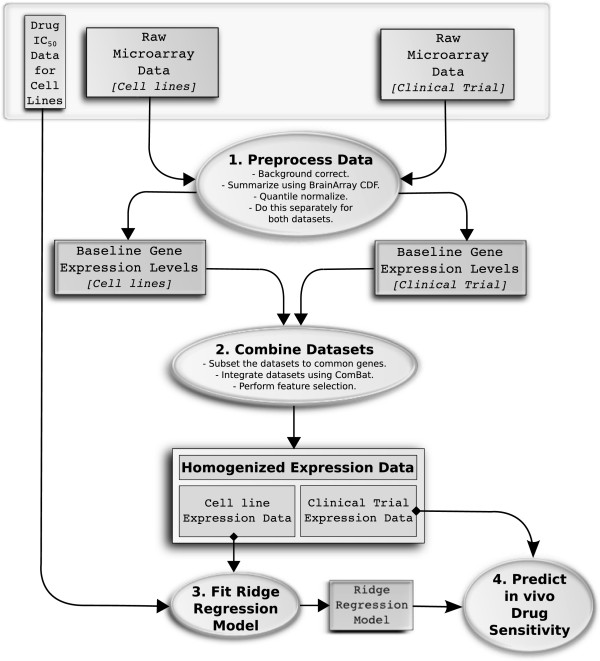
**Data flow diagram showing our approach to predicting *in vivo *drug sensitivity.** Data are represented by rectangles and processes by ovals. The input data (baseline expression and drug IC_50_ in cell lines and *in vivo* tumor gene expression) are shown in a gray box. The raw microarray data are (1) preprocessed separately using the robust multi-array average [26] method and the CDF files remapped by BrainArray [27] are summarized, (2) then combined and homogenized using ComBat. (3) A ridge regression model is fitted for baseline gene expression levels in the cell lines against the *in vitro* drug IC_50_ estimates and (4) this model is then applied to the baseline tumor expression data from the clinical trial, to yield drug sensitivity estimates. Complete details are given in Materials and methods. CDF, chip definition file.

**Figure 2:**
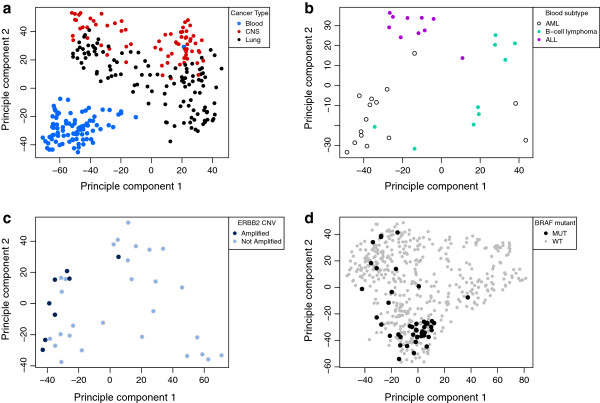
**Whole-genome gene expression data capture molecular phenotypes in cancer cell lines. (a)** Clustering of cancer types on principal component (PC) 1 and PC2 of a gene expression matrix from the CGP cell lines. There is clear clustering of blood, central nervous system and lung cancers. For clarity, only these three cancer types are shown here. Additional file 1: Figure S1 shows all cancer types. **(b)** Clustering of subtypes of hematological cancers on PC1 and PC2 of a gene expression matrix of CGP hematological cancer cell lines. For clarity only acute myeloid leukemia, acute lymphoblastic leukemia and B-cell lymphoma are shown here. All data is shown in Additional file 1: Figure S2. **(c)** Clustering of *ERBB2* amplified breast cancers on PC1 and PC2 of a gene expression matrix of CGP breast cancer cell lines. **(d)** Clustering of BRAF mutated cancers on PC1 and PC2 of a gene expression matrix from all CGP cell lines. ALL, acute lymphoblastic leukemia; AML, acute myeloid leukemia; CGP, Cancer Genome Project; CNS, central nervous system; CNV, copy number variation; MT, mutated; PC, principal component; WT, wild type.

Our goal was to use baseline gene expression and *in vitro* drug sensitivity derived from cell lines, coupled with *in vivo* baseline tumor gene expression, to predict patients’ response to drugs. An overview of our approach is shown in Figure 1 (complete details are in Materials and methods; see Data availability for details of how to acquire the R code). Ridge regression models, which allow a small contribution from every gene, have previously been shown to be the best method for predicting survival from gene expression microarray data [13,14]. Our analyses are consistent with these previously published findings. In preliminarily tests, we assessed several of the plethora of available machine learning algorithms, including random forests [21], PAM [22], principal component regression [23], Lasso [24] and ElasticNet regression [25]. Among them, ridge regression was consistently the best performer, with the added advantage of being highly computationally efficient, which is crucial for cross-validation analysis. Furthermore, principal component analysis (PCA) demonstrates that whole-genome gene expression can capture far more information about cancer biology, than may have been previously appreciated. As illustrated in Figure 2 and Additional file 1: Figures S1 and S2, whole-genome gene expression recapitulates tissue of origin, cancer subtype and various genomic aberrations, when the CGP cell lines are plotted on the first two principal components of a whole-genome gene expression matrix. This suggests that whole-genome gene expression acts as a surrogate for unmeasured genetic and non-genetic phenotypes, providing additional support for this approach.

### Docetaxel and cisplatin treatment of breast cancer

We first applied our method to gene expression microarray data obtained from 24 breast cancer tumor biopsies through a clinical trial, which measured the response of patients to docetaxel neoadjuvant treatment [18]. Tumor size, measured before and after four cycles of docetaxel, was used to calculate the percentage of residual disease. The authors designated individuals as ‘sensitive’ or ‘resistant’ to docetaxel, depending on whether there was ≤25% or >25% of the tumor remaining. Tumor gene expression levels were measured from biopsies using Affymetrix microarrays (GEO accession number [GEO:GSE6434]). In the original study, receiver operating characteristic (ROC) curve [28,29] analysis reported an area under the curve (AUC) of 0.96 (from leave-one-out cross-validation (LOOCV)) using a 92-gene signature derived from the 24 samples. However, given that this signature was generated on the same data on which it was evaluated, this is likely to represent an inflated estimate of the classification accuracy.

**Figure 3:**
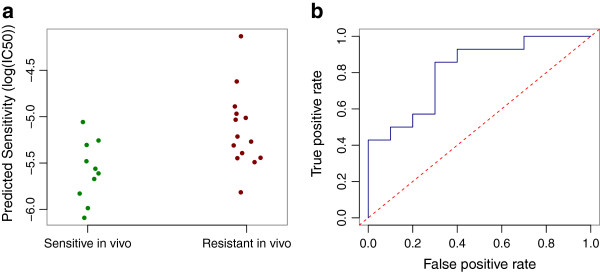
**Prediction of docetaxel sensitivity in breast cancer patients. (a)** Strip chart showing the difference in predicted drug sensitivity for individuals sensitive or resistant to docetaxel treatment *in vivo*. **(b)** ROC curve showing the proportion of true positives against the proportion of false positives as the classification threshold is varied. ROC, receiver operating characteristic.

To compare our method to these results, we used the CGP cell lines to build a ridge regression model, which related whole-genome gene expression to docetaxel sensitivity. We applied the model to the *in vivo* pretreatment breast cancer tumor expression data. The predicted drug sensitivity value was lower in the patients who were defined (by the trial) to be sensitive to docetaxel, compared to the patients defined as resistant (Figure 3a; *P* = 4.0 × 10^−3^ from *t*-test). Of the seven individuals who were predicted to be most sensitive, six are in the trial-defined sensitive group. ROC curve analysis revealed an AUC of 0.81 (Figure 3b; *P* = 5.0 × 10^−3^). Notably, training (cell lines) and test (clinical trial) data were assessed using different microarray platforms and the training set contained only 24 breast cancer cell lines. Interestingly, when the models were trained on these 24 breast cancer cell lines alone, there was no difference in predicted drug sensitivity between the trial-defined sensitive/resistant groups (*P* = 0.65 from a *t*-test). This suggests that the non-breast cancer cell lines included in the full training panel are informative for predicting the *in vivo* drug response for breast cancer. For comparison, ElasticNet and Lasso regression models were also applied to this data, but both underperformed when compared to ridge regression (*P* = 0.01 from *t*-tests for both models; Additional file 1: Table S1; see Materials and methods for details).

Next, we applied our method to a second breast cancer dataset, which assessed the response of 24 triple-negative patients to neoadjuvant cisplatin therapy [20]. We downloaded the raw data from ArrayExpress (accession number E-GEOD-18864) and processed it as described in Materials and methods. The authors assigned patients to one of four drug response categories based on RECIST [30] criteria. This time, our models did not capture variability in clinical response (Additional file 1: Figure S3; *P* = 0.26 from a linear regression model). LOOCV (see Materials and methods) indicated that, for the cell line panel, our models captured approximately the same proportion of variability in cellular response to cisplatin as they had for docetaxel (*r* = 0.35, *P* = 2.6 × 10^−15^ for docetaxel and *r* = 0.32, *P* = 1.4 × 10^−13^ for cisplatin from Pearson’s correlation test between LOOCV estimated log IC_50_ and measured log IC_50_ values). Thus, it is surprising that we could not also predict the *in vivo* response to cisplatin. Notably, the authors of the original trial could not generate a gene signature from their data, or show that any signature in the literature captured cisplatin response *in vivo*. Furthermore, they found that no genes were significantly correlated with response, following correction for multiple testing [20]. Therefore, it is possible that we (and the original authors) could not achieve statistical significance, because of the lack of variability in drug response among a small group of patients, as cisplatin is not routinely used to treat breast cancer [20]. Encouragingly, patients showing a ‘complete response’ or ‘progressive disease’ had the lowest and highest median predicted drug sensitivity values, respectively (Additional file 1: Figure S3); but given that there were only three individuals in each of these groups, it is not surprising that we did not establish significance. Consequently, a larger clinical cohort may be required to assess rigorously whether our models capture variability in cisplatin response for triple-negative breast cancer.

### Bortezomib in myeloma

Next, we applied our approach to a larger publicly available clinical phase II/III trial dataset, which assessed response to bortezomib in relapsed multiple myeloma patients [19]. In the original study, a pretreatment bone marrow aspirate was collected and enriched for tumor cells, which underwent microarray expression profiling. It was found that 168 patients had a clinically evaluable bortezomib response, which was classified as complete response (CR), partial response (PR), minimal response (MR), no change (NC) or progressive disease (PD) [19] using European Group for Bone Marrow Transplantation criteria [31]. CR, PR and MR patients were defined as responders and NC and PD patients as non-responders. Expression in tumor cells was measured using either Affymetrix Human Genome U133A or U133B arrays in triplicate. The same samples were interrogated using both A and B arrays. Data were processed using the Affymetrix MAS5.0 algorithm and the median expression value of the replicates that passed quality control was reported.

This clinical dataset presents some obstacles. Firstly, only the preprocessed data is publicly available (GEO accession number [GEO:GSE9782]). The fact that the raw microarray data are not available is problematic because the lack of standardized raw data processing likely lowers the performance of our model. Also, clinical samples were collected as part of three different clinical trials from various sites, and they were also hybridized in different batches, reflecting the types of issues that may be encountered were one to apply such a method in the clinic. Furthermore, there is only a single myeloma cell line in the training panel.

**Figure 4:**
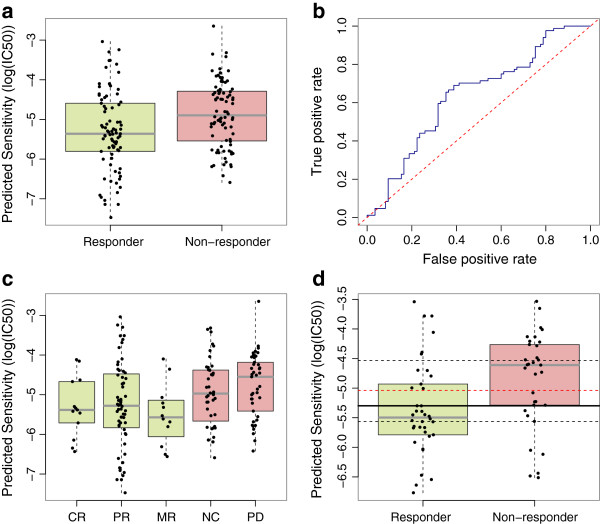
**Prediction of bortezomib sensitivity in multiple myeloma patients. (a)** Strip chart and boxplot of predicted drug sensitivity for *in vivo* responders and non-responders to bortezomib. **(b)** ROC curve illustrating estimated prediction accuracy. **(c)** Strip chart and boxplot with responders and non-responders further broken down as showing CR, PR, MR, NC or PD. **(d)** Strip chart and boxplot illustrating our predictions for the (039) arm of the bortezomib trial. The horizontal black line indicates the optimal cut-point, where classification accuracy is 72%. The horizontal dashed black lines indicate the range of cut-points for which classification accuracy is >63%, which was the accuracy reported for the trial-derived 100-gene signature on this dataset. The horizontal dashed red line is the mean IC_50_ value in the cell line training set, which could be used as an unbiased cut-point. CR, complete response; MR, minimal response; NC, no change; PD, progressive disease; PR, partial response.

LOOCV in the cell line training set revealed similar correlations to other drugs (*r* = 0.45; *P* = 2.6 × 10^−15^). Despite the suboptimal clinical data, our method captured substantial variability in bortezomib response. There was a statistically significant difference between the predicted drug sensitivity in patients between the trial-defined responder and non-responder groups (Figure 4a; *P* = 8.9 × 10^−4^ for samples quantified using U133A and Additional file 1: Figure S4; *P* = 1.5 × 10^−6^ for samples quantified using U133B from *t*-tests). In the U133A dataset, the nine patients who were predicted to be most sensitive were all drug responders (Figure 4a). The AUCs from ROC curve analysis are 0.63 and 0.71 for U133A and U133B measurements, respectively (Figure 4b; *P* = 1.3 × 10^−3^ and Additional file 1: Figure S5; *P* = 1.0 × 10^−5^). Strikingly, when the response was further subdivided (as CR, PR, MR, NC and PD), the median predicted drug sensitivity in each of these five groups was in exactly the correct order (Figure 4c and Additional file 1: Figure S6) in the U133B samples.

The authors of the original clinical study reported that a 100-gene signature model [32], built on two arms of the trial (025 and 040), could predict bortezomib response in the third (039) arm of the trial with 63% accuracy. To compare our predictions with those originally reported, we assessed the performance of our model on only this third arm of the trial. Our models generate a continuous variable and to compare the results previously reported directly, we must dichotomize this variable (i.e. split the data into ‘sensitive’ and ‘resistant’ at an arbitrary cut-point). At the optimal cut-point (−5.29), 51 of 71 patients were correctly classified, meaning that our method achieved a classification accuracy of 72%. For a large range (−5.57 to −4.53) of possible cut-points, our accuracy was greater than the 63% achieved by the trial-derived gene signature (Figure 4d). While dichotomizing clinical response data is not ideal [33], the original clinical data is again not available, thus this is the only means of directly comparing the predictions. Nevertheless, the results indicate that our models offer a substantial performance improvement.

This study also contained a group of 70 patients who were treated with dexamethasone (and had a clinically evaluable drug response). It was not possible to construct a dexamethasone specific model as this drug was not screened against the CGP cell lines. This group is still suitable as a negative control and thus we applied the bortezomib model in this cohort. Encouragingly, there was no difference in predicted bortezomib sensitivity between responders and non-responders to dexamethasone (*P* = 0.81 from a *t*-test), suggesting that the models applied to bortezomib-treated patients are drug specific.

### Erlotinib in non-small cell lung cancer

Finally, we applied our approach to a dataset from the Biomarker-Integrated Approaches of Targeted Therapy for Lung Cancer Elimination (BATTLE) study (trial registration ID: NCT00409968) [17,34]. A subset of patients with recurrent or metastatic non-small cell lung cancer (NSCLC) were treated with either erlotinib (*n* = 25), an EGFR inhibitor, or sorafenib (*n* = 37), a VEGFR inhibitor, in a second-line setting. Raw microarray and drug sensitivity data were downloaded from GEO ([GEO:GSE33072]).

Inspection of the training data revealed that only a very small proportion of the cell lines assessed for sensitivity to these drugs were within the drug screening concentration used by the CGP. This is the case for many targeted agents. In contrast, most cell lines treated with cytotoxic agents, for example docetaxel, have more accurately quantified IC_50_ values, because a much larger proportion of cell lines tended to respond within the sensitivity screening window. The drastically different response of cell lines to cytotoxic or targeted agents is illustrated in Additional file 1: Figure S7. This can be rigorously demonstrated by segmenting all drugs into two groups (cytotoxic or targeted) and comparing the median size of the confidence intervals associated with the IC_50_ values of each drug. Unsurprisingly, the confidence intervals are larger for targeted agents (average of 1.9 for cytotoxic compared to 4.5 for targeted agents; *P* = 1.4 × 10^−5^ from a Wilcoxon rank sum test). Consistent with this, the signal-to-noise ratio is also significantly different for cytotoxic and targeted drugs (*P* = 7.1 × 10^−7^ from a Wilcoxon rank sum test). In light of this, it is not reasonable to fit a linear ridge regression for most targeted agents, because IC_50_ values for most cell lines were derived using extrapolated data, and thus have very large associated confidence intervals. An approach that reduces the level of noise fitted in the model will be more suited for targeted agent sensitivity prediction. Consequently, we fitted logistic ridge regression models for the 15 most sensitive (which had reliably measured IC_50_ values) versus the 55 most resistant CGP cell lines (see Materials and methods). In LOOCV, this method provided 89% classification accuracy on the training set and separated sensitive and resistant groups with *P* = 9.3 × 10^−5^, providing additional support for applying this approach to clinical samples.

**Figure 5:**
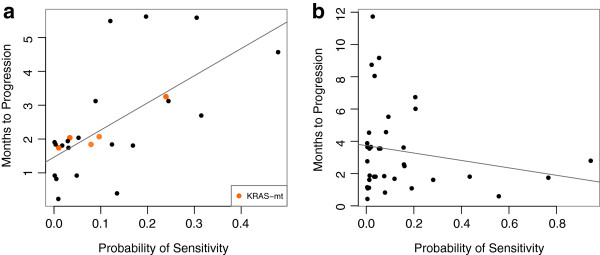
**Prediction of erlotinib sensitivity in NSCLC patients. (a)** Months-to-progression plotted against predicted probability of erlotinib sensitivity. All patients are *EGFR* wild-type. *KRAS* mutations are highlighted and a linear regression line is shown. **(b)** The predicted probability of sensitivity to erlotinib plotted against months-to-progression for individuals treated with sorafenib. NSCLC, non-small cell lung cancer.

When applied to the clinical trial data, this modified approach captured a large proportion of variability in the *in vivo* erlotinib response (Figure 5a; rho = 0.64 and *P* = 5.3 × 10^−4^ from a Spearman’s correlation test). All patients were *EGFR* wild-type. Since *KRAS* mutation is a known biomarker of resistance to EGFR inhibitors [35-37], we evaluated performance in the subset of 20 individuals who were both *EGFR* and *KRAS* wild-type. The results remained highly significant (rho = 0.59 and *P* = 6.4 × 10^−3^ from a Spearman’s correlation test). This means that we enriched for drug responders, even in the absence of any known biomarker. A further interesting observation is that one patient, who was among the most sensitive to erlotinib, had a *KRAS* mutation, normally a biomarker of EGFR inhibitor resistance. Our approach predicted this would be the fifth most sensitive individual in the cohort, and they were in fact, the fifth most sensitive individual.

The authors of the original study developed a 76-gene epithelial-mesenchymal transition (EMT) gene expression signature using both NSCLC cell lines and patient data. They reasoned, as EMT had been previously shown to be associated with EGFR inhibitor resistance, that this signature may capture variability in erlotinib response *in vivo*. The gene signature was applied to the 20 *EGFR* and *KRAS* wild-type NSCLC patients treated with erlotinib. They found that individuals with disease control at eight weeks showed a more epithelial-like signature and the result was of borderline significance (*P* = 0.052 from a *t*-test). For comparison with our approach, we assessed the difference in predicted probability of erlotinib sensitivity (from the logistic ridge regression model) between individuals with disease progression and those without disease progression at two months. In our case, the difference was highly statistically significant (*P* = 4.9 × 10^−4^ from a *t*-test). This suggests that whole-genome gene expression models, derived from a large panel of cell lines, have superior power to predict erlotinib sensitivity, compared to the 76-gene EMT signature.

Since the drug sensitivity phenotype evaluated in this trial is ‘months-to-progression’, it is possible that our models are capturing a prognostic phenotype, rather than drug sensitivity. To test this, we applied our cell-line-derived erlotinib sensitivity prediction model to predict months-to-progression for an independent arm of the same trial in which NSCLC patients were treated with sorafenib. The erlotinib-specific model was not predictive of months-to-progression after sorafenib treatment (Figure 5b; *P* = 0.83 from a *t*-test), suggesting that the model is drug specific, rather than a general predictor of disease progression or prognosis.

## Discussion

We have shown that models constructed using baseline gene expression and drug IC_50_ values, from a large panel of cell lines, can predict the chemotherapeutic response in patients. Our method uses whole-genome ridge regression, and the expression of every gene contributes a small amount to the prediction. The ridge regression penalty parameter is automatically selected, meaning that no user input is required to tune the algorithm. Our approach captures a statistically significant proportion of variability in drug response in three of four clinical trials, regardless of the drugs’ mechanism of action or the patients’ cancer type and in all cases, performance was comparable to that of the gene signatures derived directly from clinical data. To our knowledge, this is the first time that a method capable of this has been described.

It may seem surprising that whole-genome gene expression alone has such remarkable power in enriching for drug responders, as the approach ignores what are thought to be important factors in drug sensitivity, such as cancer type, genomic aberrations or any other specific markers. We showed that the impressive performance may (at least in part) stem from the fact that whole-genome gene expression may act as a surrogate for unmeasured phenotypes that are directly relevant to chemotherapeutic sensitivity (Figure 2), and capture aspects of both germ line and tumor-specific genome variation. This observation is further supported by multiple studies that have shown that gene expression can be used to characterize novel cancer subtypes and has been shown to have predictive and prognostic value [38-43].

Our method attained classification accuracy approaching, or even surpassing that of the gene signatures derived directly from clinical trials. In these trials, gene signatures were derived using *in vivo* samples. In the case of docetaxel, the signature was generated on the same set of samples on which it was tested, which inevitably inflates their estimate of prediction accuracy. It would only be possible to compare performance fairly using an independent dataset. Nevertheless, our method significantly enriched for docetaxel responders in the trial dataset. Our approach predicted *in vivo* drug sensitivity more accurately than a 100-gene signature derived from the bortezomib clinical trial. We also outperformed the 76-gene EMT signature (generated using both cell lines and patients) in predicting sensitivity to erlotinib. There was no evidence that KRAS mutation, previously identified as a biomarker of EGFR inhibitor resistance, had predictive power in this data, although the number of samples was small and this result does not discount KRAS mutation as a biomarker for this drug. The fact remains that our method also outperformed this (already established) biomarker in this dataset. We modified the original algorithm (to use logistic instead of linear ridge regression) in the analysis of erlotinib data. This is justifiable given the severe noise associated with the IC_50_ values for these types of targeted agents in the CGP cell lines. Overall, the results suggest that models created on very large panels of cell lines, can rival, or even surpass the performance of similar *in vivo* approaches.

In all studies discussed here, the data are suboptimal, because all of the clinical trials used a different microarray platform than was used on the cell line panel training set. Also, the cell line panel often only contained a very small number of samples from the actual cancer type that the clinical trial evaluated. For example, only one myeloma cell line was treated with bortezomib in our panel of training cell lines. We anticipate that if training and test microarray platforms were the same and if the cell line panel contained more relevant cancer types, accuracy would be further improved. These results are congruent with the emerging view that -omics characterization of tumors may rival traditional tissue-of-origin and pathological descriptors for a variety of clinically important classifications. This was supported by our finding that, unlike the full cell line panel, the 24 available breast cancer cell lines could not predict docetaxel response for breast cancer patients (although it would be difficult to achieve accurate prediction using such a small training set).

Performance would also likely be enhanced by a more detailed assessment of the transcriptome, for example, by quantifying transcript expression levels with RNA sequencing (RNA-seq), which has been shown to provide better estimates of expression than microarrays [44]. A recent study has also found that results of transcriptome sequencing using RNA-seq were highly reproducible between different laboratories, if procedures are standardized (which is not generally the case for expression microarrays) [45]. This provides further evidence that incorporating RNA-seq would increase power and the widespread utility of these types of expression-based prediction assays. However, a different machine learning algorithm may be better suited to the distribution of RNA-seq data.

We have recently completed a separate study that provides additional support for emphasizing transcriptomics in pharmacogenomic prediction. In that analysis, we used whole-genome models, similar to the ridge regression models used here, to compare the relative contribution of whole-genome SNPs, gene expression or microRNA (miRNA) expression, to inter-individual variability in cellular growth rate [46]. We found that, in lymphoblastoid cell lines, far more of the variability in growth rate (between cell lines isolated from different individuals) can be explained by the transcriptome than by genome-wide SNPs. Using gene and miRNA expression data, we constructed statistical models that explained 48% of variability in growth rate, compared to just 2% when using models based on only whole-genome SNP data. Given that a substantial proportion of chemotherapeutic agents target fast-growing cells (for example, docetaxel), these results provide a strong rationale for prioritizing the transcriptome when predicting clinical response to this class of drugs. The result also showed that combining miRNA and gene expression data significantly improved prediction (from 38% to 48% of variability explained) over mRNA expression alone, suggesting that including miRNA and other non-coding RNAs may improve the prediction of clinical drug sensitivity, although no data is currently available that would allow us to test this hypothesis.

Here, we have demonstrated that models derived from a very large panel of cell lines achieve equal or better performance for clinical drug sensitivity prediction than those derived directly from patients and these findings can have a profound impact on patient care. For example, screening for drug sensitivity *in vitro* is far less costly and time-consuming than conducting large clinical trials. A much larger number of samples can be screened against any given drug using cell lines than would be feasible (either practically or ethically) in a clinical setting. Furthermore, *in vitro* drug sensitivity screening is usually conducted under controlled experimental conditions to achieve a greater degree of accuracy. The fact that many more samples can be screened, with more accuracy, may lead to improved statistical power, compared to *in vivo* methods, which inevitably rely on smaller sample sizes and noisy clinical response phenotypes. This, and the fact that recently developed statistical methods robustly correct for intrinsic differences in gene expression between cell lines and *in vivo* tumors, suggest that this type of approach is a very promising option for personalizing drug treatment. There is no limit to the number of drugs that could be screened against a panel of cell lines. In theory, every existing chemotherapeutic compound could be tested, meaning that given a tumor biopsy, it would be trivial to use this approach to estimate sensitivity to every drug prior to any course of treatment. The falling price of gene expression microarrays makes it feasible to incorporate this technology into patient care. The cell line training set could also be expanded by including data on cellular sensitivity within different microenvironments and cell lines under different simulated stromal conditions, as it is known that the tumor microenvironment plays a key role in tumor development [47].

The results also have important implications in drug development, where our approach could be used to enrich for likely drug responders, prior to carrying out clinical trials. There has been much recent interest in developing drugs in conjunction with companion diagnostic tests [48]. The benefits of enriching for likely drug responders are obvious, but it is normally difficult to develop accurate biomarkers without exposing the drug to patients. However, our approach, because of its ability to enrich for drug responders in a clinical cohort, has enormous potential as a companion diagnostic. There is also a clear ethical benefit, in that such a diagnostic could be developed without ever exposing potentially unresponsive patients to toxic chemotherapeutic agents.

Finally, we highlight the work of Menden *et al.*, who recently constructed models using the CGP cell line data, with the aim of predicting *in vitro* drug sensitivity [49]. The authors achieved impressive prediction (*R*^2^ = 0.72 from eightfold cross-validation) by using models that consider the effectiveness of drugs with similar mechanisms of action. These results are better than those achieved using our models (based on expression data alone), but currently cannot be extended to *in vivo* data because clinical response to similar drugs is almost never known *a priori*. However, the results suggest that in future, *in vivo* prediction could be improved by considering multiple drugs, using either prior information on what is known about mechanism of drug action, or simply the empirical correlation of drug sensitivities.

## Conclusions

In summary, we have shown for the first time that it is possible to enrich for drug responders in a clinical cohort using only baseline tumor gene expression levels, by applying models generated from a large panel of cell lines. We have also demonstrated that this approach outperforms several existing biomarkers in the available clinical datasets. These findings have profound implications for personalized medicine and drug development. Future work will focus on improving predictions using more rigorous transcriptome quantification and further testing in prospective clinical trials. The R code [50] needed to reproduce all figures and results in this paper, is provided (for academic use) on our website in Sweave [51] format (see Data availability).

## Materials and methods

### Bioinformatics analysis overview

Bioinformatics analyses were performed in R. Our implementation is extremely fast (typically running in <30 s on a standard desktop computer) and easy to use. Once the data are correctly loaded, the user need only provide the baseline gene expression and drug sensitivity data (i.e. IC_50_) from the cell line panel and baseline gene expression data from the clinical trial. The predicted drug sensitivity is then calculated, requiring no further user input. All R code is provided in annotated Sweave format on our website (see Data availability), enabling other investigators to reproduce easily all the results and figures presented in this paper.

### Obtaining gene expression and drug sensitivity data

Drug IC_50_ values for docetaxel, bortezomib and erlotinib were downloaded from the CGP website ([52]; accessed August 2013). The raw CGP gene expression microarray data (CEL files) were downloaded from ArrayExpress under accession number E-MTAB-783. These data were preprocessed using the robust multi-array average algorithm (implemented by the rma() function in the affy [53] library in R). This algorithm does background correction, quantile normalization and median-polish summarization in one step. For summarization, we used the updated probeset annotation chip definition file (CDF) provided by BrainArray (version 17.0.0 for Affymetrix HT Human Genome U133A arrays, probesets mapped to Entrez gene IDs). We followed the same set of steps to preprocess the docetaxel, cisplatin and erlotinib/sorafenib clinical trial gene expression data (using the appropriate BrainArray CDF file in each case). The bortezomib expression data were obtained directly from GEO using the getGEO() function implemented in the R library GEOquery [54]*.* All *in vivo* drug response data were obtained from GEO or directly from the relevant publication.

### Combining and homogenizing cell line and clinical trial gene expression datasets

Training (cell lines) and test (clinical trial) datasets were mapped to official gene symbols. Probesets that mapped to more than one gene symbol were summarized by their mean expression value. In all cases, both datasets were generated on different microarray platforms, thus we used a subset of genes represented on both platforms. This typically left approximately 10,000 gene symbols remaining. These two datasets were then homogenized using the ComBat() function from the sva library in R. Finally, we filtered out genes whose expression did not vary substantially in the homogenized dataset, because if technical variability is greater than biological variability, these can never add predictive value; we removed the 20% of genes with lowest variability in expression across all samples.

### Predicting *in vivo* drug sensitivity using linear ridge regression for docetaxel, cisplatin and bortezomib clinical trials

Once the data were prepared as outlined above, a linear ridge regression model was fitted for *in vitro* drug sensitivity dependent on the homogenized whole-genome expression levels in the CGP cell lines (for which both drug sensitivity and expression data were available). To do this, we used the linearRidge() function from the ridge [55] package in R. This function implements a method to choose the ridge regression tuning parameter automatically. Before fitting the model, the drug sensitivity phenotype data (IC_50_ values) were power transformed using the powerTransform() function in the R package car. After the model was fitted, it was then applied to the homogenized gene expression data from the clinical trial, using the predict.linearRidge() function from the ridge package in R, thus yielding a drug sensitivity estimate for each patient.

### Leave-one-out cross-validation

For LOOCV, all data were first preprocessed and homogenized as described above. Then, ridge regression models were fitted (as above) on all of the available cell line data, but with one sample omitted. Next, these models were used to calculate a predicted IC_50_ value, using the gene expression data of the single omitted cell line. This process was repeated iteratively for every sample thus yielding a predicted IC_50_ for every cell line. These predicted IC_50_ values were then compared to the measured IC_50_ values, using a Pearson’s correlation test, giving an estimate of prediction accuracy.

### ElasticNet and Lasso models

ElasticNet and Lasso regression models were fitted using the glmnet package in R. The Lasso penalty parameter was selected using the automatic cross-validation feature (i.e. the cv.glmnet() function). ElasticNet penalty parameters (alpha and lambda) were selected using the caret package in R. Optimal parameters were selected using a grid search on the cell line training set, which takes approximately one day to run on a standard PC. Parameters were selected based on an optimal *R*^2^ value or Cohen’s kappa (in cross-validation) for linear and logistic models, respectively.

### Predicting *in vivo* drug sensitivity using logistic ridge regression for an erlotinib clinical trial

The data were first prepared as described above. Next, we divided the cell line training data into sensitive (15 samples) or resistant (55 samples) groups and fitted a logistic ridge regression model using the logisticRidge() function from the R package ridge. Again, the ridge regression tuning parameter was automatically selected. As this implementation of logistic ridge regression is extremely computationally intensive, we implemented a feature selection step, where only the 1,000 genes that were most differentially expressed between the 15 sensitive and 55 resistant samples were fitted in the model. These genes were selected using *t*-tests, specifically using the rowttests() function in the R library genefilter [56]*.* This step enables a standard desktop computer to fit a model in approximately ten minutes (as opposed to days). Once the model is fitted, it is applied to the homogenized gene expression data from the clinical trial, using the predict.logisticRidge() function, which calculates the predicted log-odds of drug sensitivity.

### Statistical analysis of results

ROC curve analysis was performed using the ROCR [28] package in R. Empirical *P-*values were generated using 100,000 sample label permutations and computing the proportion of permutations for which the AUC was more extreme than that observed in the original data. Linear regression, *t*-tests and Spearman’s correlation tests were performed using the base functions in R. Figure 1 was generated using the Inkscape software and the remaining figures were generated in R.

### Data availability

Annotated R code (in Sweave format) to reproduce all of the analysis in this paper is available from our website [57]. The CGP gene expression data are available from ArrayExpress under accession number E-MTAB-783. The IC_50_ data for the drugs is available from the CGP website [52]. The docetaxel data are available from GEO under accession numbers [GEO:GSE349] and [GEO:GSE350]. The cisplatin data are available from ArrayExpress under accession number E-GEOD-18864*.* The bortezomib data are available from GEO under accession number [GEO:GSE9782]. The erlotinib data are available from GEO under accession number [GEO:GSE33072]. Complete details and R code showing how to acquire and preprocess all of these data, as well as the associated clinical data are available in Sweave format on our website.

## Abbreviations

AUC: area under the curve; CDF: chip definition file; CGP: Cancer Genome Project; CNS: central nervous system; CR: complete response; EMT: epithelial-mesenchymal transition; LOOCV: leave-one-out cross-validation; miRNA: microRNA; MR: minimal response; NC: no change; NSCLC: non-small cell lung cancer; PCA: principal component analysis; PD: progressive disease; PR: partial response; RNA-seq: RNA sequencing; ROC: receiver operating characteristic; SNP: single nucleotide polymorphism.

## Competing interests

The authors declare that they have no competing interests.

## Authors’ contributions

PG developed the method, performed the analysis and drafted the paper. RSH initiated and supervised the project. NJC assisted in supervising the project. All authors edited and approved the final manuscript.

## Supplementary Material

Additional file 1 PDF file containing all supplementary figures, tables and their associated legends. Click here for file

## References

[B1] Mishra A Verma M Cancer biomarkers: are we ready for the prime time? Cancers (Basel) 2010 2 190 208 10.3390/cancers2010190 24281040 PMC3827599

[B2] Jiang Y Wang M Personalized medicine in oncology: tailoring the right drug to the right patient Biomark Med 2010 4 523 533 10.2217/bmm.10.66 20701441

[B3] Simon R Roychowdhury S Implementing personalized cancer genomics in clinical trials Nat Rev Drug Discov 2013 12 358 369 10.1038/nrd3979 23629504

[B4] Sawyers CL The cancer biomarker problem Nature 2008 452 548 552 10.1038/nature06913 18385728

[B5] Lee SH Van Der Werf JHJ Hayes BJ Goddard ME Visscher PM Predicting unobserved phenotypes for complex traits from whole-genome SNP data PLoS Genet 2008 4 e1000231 10.1371/journal.pgen.1000231 18949033 PMC2565502

[B6] Ober U Ayroles JF Stone EA Richards S Zhu D Gibbs RA Stricker C Gianola D Schlather M Mackay TFC Simianer H Using whole-genome sequence data to predict quantitative trait phenotypes in *Drosophila melanogaster* PLoS Genet 2012 8 e1002685 10.1371/journal.pgen.1002685 22570636 PMC3342952

[B7] Adey A Burton JN Kitzman JO Hiatt JB Lewis AP Martin BK Qiu R Lee C Shendure J The haplotype-resolved genome and epigenome of the aneuploid HeLa cancer cell line Nature 2013 500 207 211 10.1038/nature12064 23925245 PMC3740412

[B8] Van de Vijver MJ He YD van’t Veer LJ Dai H Hart AAM Voskuil DW Schreiber GJ Peterse JL Roberts C Marton MJ Parrish M Atsma D Witteveen A Glas A Delahaye L van der Velde T Bartelink H Rodenhuis S Rutgers ET Friend SH Bernards R A gene-expression signature as a predictor of survival in breast cancer N Engl J Med 2002 347 1999 2009 10.1056/NEJMoa021967 12490681

[B9] Sotiriou C Wirapati P Loi S Harris A Fox S Smeds J Nordgren H Farmer P Praz V Haibe-Kains B Desmedt C Larsimont D Cardoso F Peterse H Nuyten D Buyse M Van de Vijver MJ Bergh J Piccart M Delorenzi M Gene expression profiling in breast cancer: understanding the molecular basis of histologic grade to improve prognosis J Natl Cancer Inst 2006 98 262 272 10.1093/jnci/djj052 16478745

[B10] Shi L Campbell G Jones WD Campagne F Wen Z Walker SJ Su Z Chu T-M Goodsaid FM Pusztai L Shaughnessy JD Oberthuer A Thomas RS Paules RS Fielden M Barlogie B Chen W Du P Fischer M Furlanello C Gallas BD Ge X Megherbi DB Symmans WF Wang MD Zhang J Bitter H Brors B Bushel PR Bylesjo M The MicroArray Quality Control (MAQC)-II study of common practices for the development and validation of microarray-based predictive models Nat Biotechnol 2010 28 827 838 10.1038/nbt.1665 20676074 PMC3315840

[B11] Shi L Reid LH Jones WD Shippy R Warrington JA Baker SC Collins PJ de Longueville F Kawasaki ES Lee KY Luo Y Sun YA Willey JC Setterquist RA Fischer GM Tong W Dragan YP Dix DJ Frueh FW Goodsaid FM Herman D Jensen RV Johnson CD Lobenhofer EK Puri RK Schrf U Thierry-Mieg J Wang C Wilson M Wolber PK The MicroArray Quality Control (MAQC) project shows inter- and intraplatform reproducibility of gene expression measurements Nat Biotechnol 2006 24 1151 1161 10.1038/nbt1239 16964229 PMC3272078

[B12] Rudy J Valafar F Empirical comparison of cross-platform normalization methods for gene expression data BMC Bioinformatics 2011 12 467 10.1186/1471-2105-12-467 22151536 PMC3314675

[B13] Van Wieringen WN Kun D Hampel R Boulesteix A-L Survival prediction using gene expression data: a review and comparison Comput Stat Data Anal 2009 53 1590 1603 10.1016/j.csda.2008.05.021

[B14] Bøvelstad HM Nygård S Størvold HL Aldrin M Borgan Ø Frigessi A Lingjaerde OC Predicting survival from microarray data – a comparative study Bioinformatics 2007 23 2080 2087 10.1093/bioinformatics/btm305 17553857

[B15] Garnett MJ Edelman EJ Heidorn SJ Greenman CD Dastur A Lau KW Greninger P Thompson IR Luo X Soares J Liu Q Iorio F Surdez D Chen L Milano RJ Bignell GR Tam AT Davies H Stevenson JA Barthorpe S Lutz SR Kogera F Lawrence K McLaren-Douglas A Mitropoulos X Mironenko T Thi H Richardson L Zhou W Jewitt F Systematic identification of genomic markers of drug sensitivity in cancer cells Nature 2012 483 570 575 10.1038/nature11005 22460902 PMC3349233

[B16] Barretina J Caponigro G Stransky N Venkatesan K Margolin AA Kim S Wilson CJ Lehár J Kryukov GV Sonkin D Reddy A Liu M Murray L Berger MF Monahan JE Morais P Meltzer J Korejwa A Jané-Valbuena J Mapa FA Thibault J Bric-Furlong E Raman P Shipway A Engels IH Cheng J Yu GK Yu J Aspesi P de Silva M The cancer cell line encyclopedia enables predictive modelling of anticancer drug sensitivity Nature 2012 483 603 607 10.1038/nature11003 22460905 PMC3320027

[B17] Byers LA Diao L Wang J Saintigny P Girard L Peyton M Shen L Fan Y Giri U Tumula PK Nilsson MB Gudikote J Tran H Cardnell RJG Bearss DJ Warner SL Foulks JM Kanner SB Gandhi V Krett N Rosen ST Kim ES Herbst RS Blumenschein GR Lee JJ Lippman SM Ang KK Mills GB Hong WK Weinstein JN An epithelial-mesenchymal transition gene signature predicts resistance to EGFR and PI3K inhibitors and identifies Axl as a therapeutic target for overcoming EGFR inhibitor resistance Clin Cancer Res 2013 19 279 290 10.1158/1078-0432.CCR-12-1558 23091115 PMC3567921

[B18] Chang JC Wooten EC Tsimelzon A Hilsenbeck SG Gutierrez MC Elledge R Mohsin S Osborne CK Chamness GC Allred DC O’Connell P Gene expression profiling for the prediction of therapeutic response to docetaxel in patients with breast cancer Lancet 2003 362 362 369 10.1016/S0140-6736(03)14023-8 12907009

[B19] Mulligan G Mitsiades C Bryant B Zhan F Chng WJ Roels S Koenig E Fergus A Huang Y Richardson P Trepicchio WL Broyl A Sonneveld P Shaughnessy JD Bergsagel PL Schenkein D Esseltine D-L Boral A Anderson KC Gene expression profiling and correlation with outcome in clinical trials of the proteasome inhibitor bortezomib Blood 2007 109 3177 3188 10.1182/blood-2006-09-044974 17185464

[B20] Silver DP Richardson AL Eklund AC Wang ZC Szallasi Z Li Q Juul N Leong C-O Calogrias D Buraimoh A Fatima A Gelman RS Ryan PD Tung NM De Nicolo A Ganesan S Miron A Colin C Sgroi DC Ellisen LW Winer EP Garber JE Efficacy of neoadjuvant cisplatin in triple-negative breast cancer J Clin Oncol 2010 28 1145 1153 10.1200/JCO.2009.22.4725 20100965 PMC2834466

[B21] Breiman L Random forests Mach Learn 2001 45 5 32 10.1023/A:1010933404324

[B22] Tibshirani R Hastie T Narasimhan B Chu G Diagnosis of multiple cancer types by shrunken centroids of gene expression Proc Natl Acad Sci USA 2002 99 6567 6572 10.1073/pnas.082099299 12011421 PMC124443

[B23] Jolliffe IT A note on the use of principal components in regression Appl Stat 1982 31 300 10.2307/2348005

[B24] Tibshirani R Regression shrinkage and selection via the Lasso J R Stat Soc 1994 58 267 288

[B25] Hui Zou TH Regularization and variable selection via the elastic net J R Stat Soc 2005 67 301 320 10.1111/j.1467-9868.2005.00503.x

[B26] Irizarry RA Hobbs B Collin F Beazer-Barclay YD Antonellis KJ Scherf U Speed TP Exploration, normalization, and summaries of high density oligonucleotide array probe level data Biostatistics 2003 4 249 264 10.1093/biostatistics/4.2.249 12925520

[B27] Dai M Wang P Boyd AD Kostov G Athey B Jones EG Bunney WE Myers RM Speed TP Akil H Watson SJ Meng F Evolving gene/transcript definitions significantly alter the interpretation of GeneChip data Nucleic Acids Res 2005 33 e175 10.1093/nar/gni179 16284200 PMC1283542

[B28] Sing T Sander O Beerenwinkel N Lengauer T ROCR: visualizing classifier performance in R Bioinformatics 2005 21 3940 3941 10.1093/bioinformatics/bti623 16096348

[B29] Fawcett T ROC Graphs: Notes and Practical Considerations for Researchers 2004

[B30] Eisenhauer EA Therasse P Bogaerts J Schwartz LH Sargent D Ford R Dancey J Arbuck S Gwyther S Mooney M Rubinstein L Shankar L Dodd L Kaplan R Lacombe D Verweij J New response evaluation criteria in solid tumours: revised RECIST guideline (version 1.1) Eur J Cancer 2009 45 228 247 10.1016/j.ejca.2008.10.026 19097774

[B31] Bladé J Samson D Reece D Apperley J Björkstrand B Gahrton G Gertz M Giralt S Jagannath S Vesole D Criteria for evaluating disease response and progression in patients with multiple myeloma treated by high-dose therapy and haemopoietic stem cell transplantation. Myeloma Subcommittee of the EBMT. European Group for Blood and Marrow Transplant Br J Haematol 1998 102 1115 1123 10.1046/j.1365-2141.1998.00930.x 9753033

[B32] Wright G Tan B Rosenwald A Hurt EH Wiestner A Staudt LM A gene expression-based method to diagnose clinically distinct subgroups of diffuse large B cell lymphoma Proc Natl Acad Sci USA 2003 100 9991 9996 10.1073/pnas.1732008100 12900505 PMC187912

[B33] Royston P Altman DG Sauerbrei W Dichotomizing continuous predictors in multiple regression: a bad idea Stat Med 2006 25 127 141 10.1002/sim.2331 16217841

[B34] Kim ES Herbst RS Wistuba II Lee JJ Blumenschein GR Tsao A Stewart DJ Hicks ME Erasmus J Gupta S Alden CM Liu S Tang X Khuri FR Tran HT Johnson BE Heymach JV Mao L Fossella F Kies MS Papadimitrakopoulou V Davis SE Lippman SM Hong WK The BATTLE trial: personalizing therapy for lung cancer Cancer Discov 2011 1 44 53 10.1158/2159-8274.CD-10-0010 22586319 PMC4211116

[B35] Eberhard DA Johnson BE Amler LC Goddard AD Heldens SL Herbst RS Ince WL Jänne PA Januario T Johnson DH Klein P Miller VA Ostland MA Ramies DA Sebisanovic D Stinson JA Zhang YR Seshagiri S Hillan KJ Mutations in the epidermal growth factor receptor and in KRAS are predictive and prognostic indicators in patients with non-small-cell lung cancer treated with chemotherapy alone and in combination with erlotinib J Clin Oncol 2005 23 5900 5909 10.1200/JCO.2005.02.857 16043828

[B36] Paez JG Jänne PA Lee JC Tracy S Greulich H Gabriel S Herman P Kaye FJ Lindeman N Boggon TJ Naoki K Sasaki H Fujii Y Eck MJ Sellers WR Johnson BE Meyerson M EGFR mutations in lung cancer: correlation with clinical response to gefitinib therapy Science 2004 304 1497 1500 10.1126/science.1099314 15118125

[B37] Lynch TJ Bell DW Sordella R Gurubhagavatula S Okimoto RA Brannigan BW Harris PL Haserlat SM Supko JG Haluska FG Louis DN Christiani DC Settleman J Haber DA Activating mutations in the epidermal growth factor receptor underlying responsiveness of non-small-cell lung cancer to gefitinib N Engl J Med 2004 350 2129 2139 10.1056/NEJMoa040938 15118073

[B38] Zhan F Huang Y Colla S Stewart JP Hanamura I Gupta S Epstein J Yaccoby S Sawyer J Burington B Anaissie E Hollmig K Pineda-Roman M Tricot G van Rhee F Walker R Zangari M Crowley J Barlogie B Shaughnessy JD The molecular classification of multiple myeloma Blood 2006 108 2020 2028 10.1182/blood-2005-11-013458 16728703 PMC1895543

[B39] Agnelli L Bicciato S Mattioli M Fabris S Intini D Verdelli D Baldini L Morabito F Callea V Lombardi L Neri A Molecular classification of multiple myeloma: a distinct transcriptional profile characterizes patients expressing CCND1 and negative for 14q32 translocations J Clin Oncol 2005 23 7296 7306 10.1200/JCO.2005.01.3870 16129847

[B40] Tan IB Ivanova T Lim KH Ong CW Deng N Lee J Tan SH Wu J Lee MH Ooi CH Rha SY Wong WK Boussioutas A Yeoh KG So J Yong WP Tsuburaya A Grabsch H Toh HC Rozen S Cheong JH Noh SH Wan WK Ajani JA Lee J-S Tellez MS Tan P Intrinsic subtypes of gastric cancer, based on gene expression pattern, predict survival and respond differently to chemotherapy Gastroenterology 2011 141 476–85 485 e1–11 21684283 10.1053/j.gastro.2011.04.042 PMC3152688

[B41] Bertucci F Finetti P Rougemont J Charafe-Jauffret E Cervera N Tarpin C Nguyen C Xerri L Houlgatte R Jacquemier J Viens P Birnbaum D Gene expression profiling identifies molecular subtypes of inflammatory breast cancer Cancer Res 2005 65 2170 2178 10.1158/0008-5472.CAN-04-4115 15781628

[B42] Marisa L de Reyniès A Duval A Selves J Gaub MP Vescovo L Etienne-Grimaldi M-C Schiappa R Guenot D Ayadi M Kirzin S Chazal M Fléjou J-F Benchimol D Berger A Lagarde A Pencreach E Piard F Elias D Parc Y Olschwang S Milano G Laurent-Puig P Boige V Gene expression classification of colon cancer into molecular subtypes: characterization, validation, and prognostic value PLoS Med 2013 10 e1001453 10.1371/journal.pmed.1001453 23700391 PMC3660251

[B43] Lapointe J Li C Higgins JP van de Rijn M Bair E Montgomery K Ferrari M Egevad L Rayford W Bergerheim U Ekman P DeMarzo AM Tibshirani R Botstein D Brown PO Brooks JD Pollack JR Gene expression profiling identifies clinically relevant subtypes of prostate cancer Proc Natl Acad Sci USA 2004 101 811 816 10.1073/pnas.0304146101 14711987 PMC321763

[B44] Xu X Zhang Y Williams J Antoniou E McCombie WR Wu S Zhu W Davidson NO Denoya P Li E Parallel comparison of Illumina RNA-Seq and Affymetrix microarray platforms on transcriptomic profiles generated from 5-aza-deoxy-cytidine treated HT-29 colon cancer cells and simulated datasets BMC Bioinformatics 2013 14 S1 23902433 10.1186/1471-2105-14-S9-S1 PMC3697991

[B45] ’t Hoen PAC Friedländer MR Almlöf J Sammeth M Pulyakhina I Anvar SY Laros JFJ Buermans HPJ Karlberg O Brännvall M van Ommen G-JB Estivill X Guigó R Syvänen A-C Gut IG Dermitzakis ET Antonorakis SE Brazma A Flicek P Schreiber S Rosenstiel P Meitinger T Strom TM Lehrach H Sudbrak R Carracedo A van Iterson M Monlong J Lizano E Bertier G Reproducibility of high-throughput mRNA and small RNA sequencing across laboratories Nat Biotechnol 2013 31 1015 1022 10.1038/nbt.2702 24037425

[B46] Wheeler HE Aquino-Michaels K Gamazon ER Trubetskoy VV Dolan ME Huang RS Cox NJ Im HK Poly-omic prediction of complex traits: OmicKriging 2013 10.1002/gepi.21808 PMC4072756 24799323

[B47] Bremnes RM Dønnem T Al-Saad S Al-Shibli K Andersen S Sirera R Camps C Marinez I Busund L-T The role of tumor stroma in cancer progression and prognosis: emphasis on carcinoma-associated fibroblasts and non-small cell lung cancer J Thorac Oncol 2011 6 209 217 10.1097/JTO.0b013e3181f8a1bd 21107292

[B48] Zhang CH Zhang YP Maximizing the commercial value of personalized therapeutics and companion diagnostics Nat Biotechnol 2013 31 803 805 10.1038/nbt.2679 24022154

[B49] Menden MP Iorio F Garnett M McDermott U Benes CH Ballester PJ Saez-Rodriguez J Machine learning prediction of cancer cell sensitivity to drugs based on genomic and chemical properties PLoS One 2013 8 e61318 10.1371/journal.pone.0061318 23646105 PMC3640019

[B50] Team RDC R: A Language and Environment for Statistical Computing 2008 Austria, Vienna

[B51] Leisch F Dynamic generation of statistical reports using literate data analysis Proc Comput Stat 2002 575 580 http://link.springer.com/chapter/10.1007%2F978-3-642-57489-4_89#

[B52] The cancer genome project http://www.cancerrxgene.org

[B53] Gautier L Cope L Bolstad BM Irizarry RA affy–analysis of Affymetrix GeneChip data at the probe level Bioinformatics 2004 20 307 315 10.1093/bioinformatics/btg405 14960456

[B54] Davis S Meltzer PS GEOquery: a bridge between the Gene Expression Omnibus (GEO) and BioConductor Bioinformatics 2007 23 1846 1847 10.1093/bioinformatics/btm254 17496320

[B55] Cule E De Iorio M Ridge regression in prediction problems: automatic choice of the ridge parameter Genet Epidemiol 2013 37 704 714 10.1002/gepi.21750 23893343 PMC4377081

[B56] Gentleman R Carey V Huber W Hahne F genefilter: methods for filtering genes from microarray experiments http://cobra20.fhcrc.org/packages/release/bioc/html/genefilter.html

[B57] University of Chicago GeneMed Server http://genemed.uchicago.edu/~pgeeleher/cgpPrediction/

