# Supplementary material for: Clinical drug response can be predicted using baseline gene expression levels and in vitro drug sensitivity in cell lines
Source: Genome Biol. 2014 Mar 3;15(3):R47. doi: 10.1186/gb-2014-15-3-r47 (PMC4054092; doi:10.1186/gb-2014-15-3-r47)
Supplement: Additional file 1 — PDF file containing all supplementary figures, tables and their associated legends. [file gb-2014-15-3-r47-S1.pdf]

Supplementary Figures and Tables

January 13, 2014

Figures in this file are best viewed on a computer, because there is a lot of detail in some of the graphics, which may be difficult to distinguish in a print-out. On a computer, one can zoom-in indefinitely without loss of picture quality (as these are all vector graphics). This applies to all plots in this file.

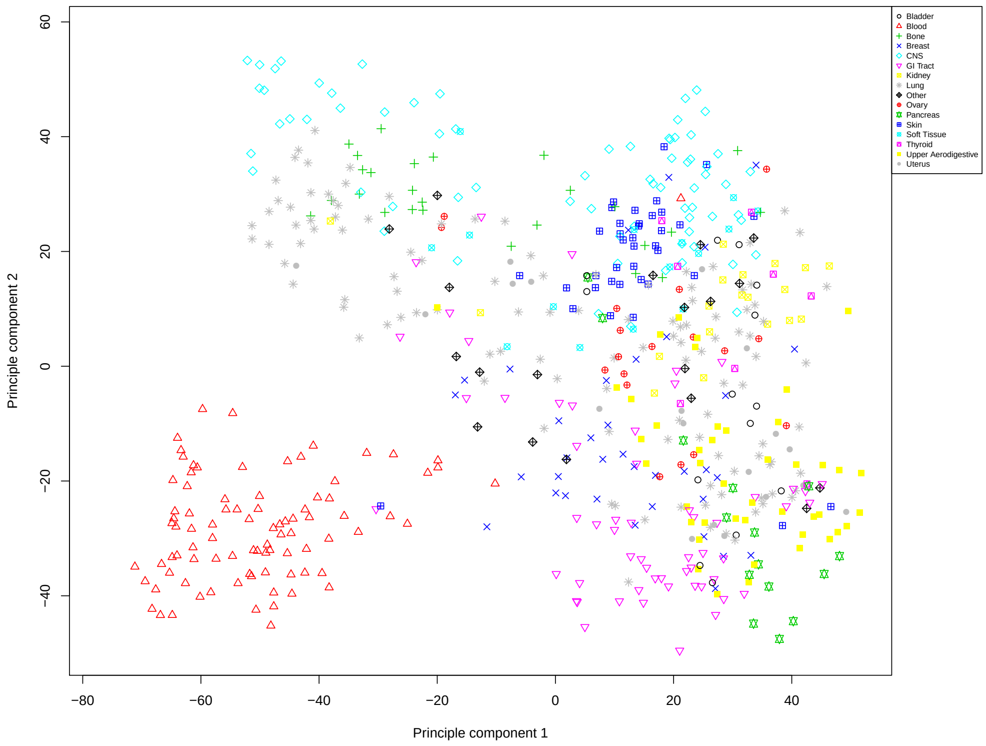

Figure 1: Clustering of all cancer types on principle component (PC) 1 and PC2 of a gene expression matrix from the GDSC cell lines.

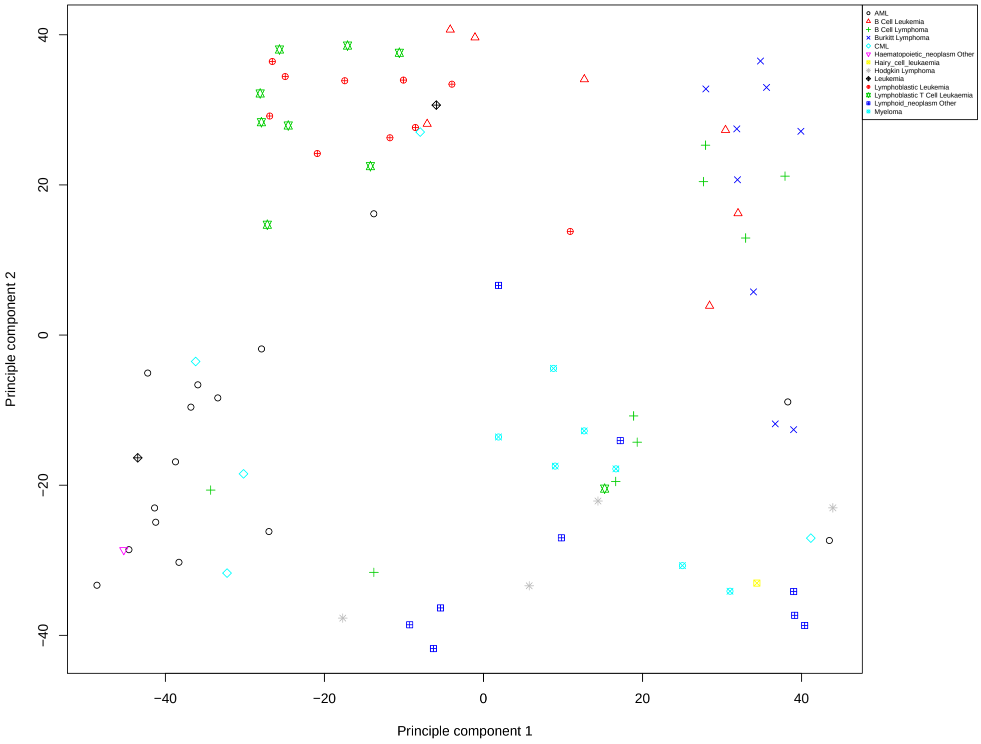

Figure 2: Clustering of subtypes of haematological cancers on PC1 and PC2 of a gene expression matrix of GDSC haematological cancer cell lines.

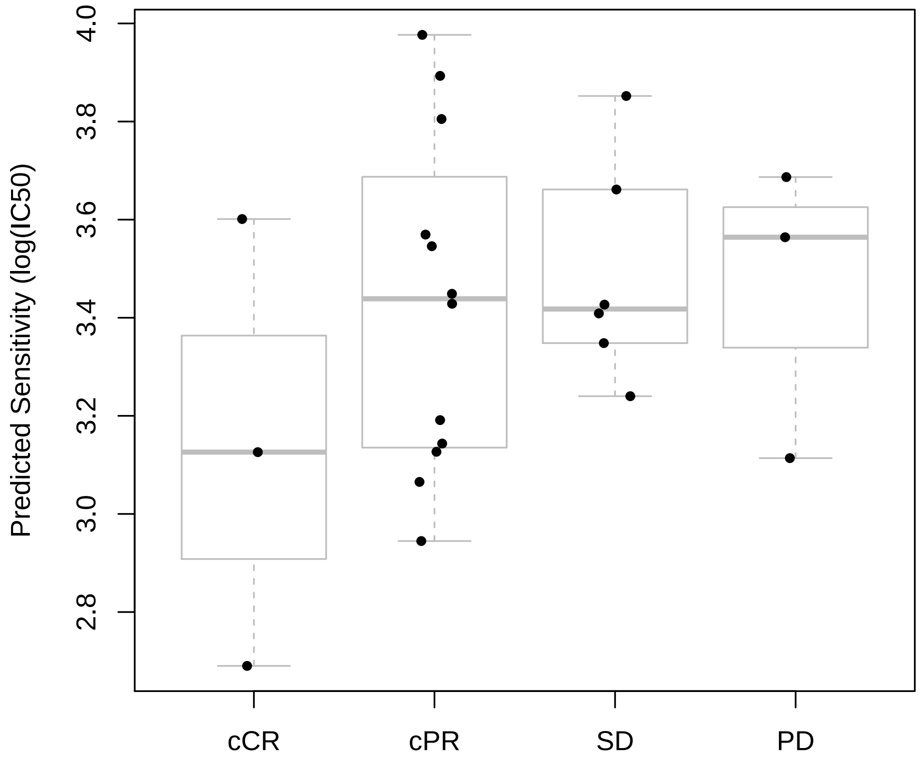

Figure 3: The predicted cisplatin sensitivity for 24 triple negative breast cancer patients for whom gene expression microarray and clinical response data were available. Clinical response are categorized as "clinical complete response" (cCR), "clinical partial response" (cPR), "stable disease" (SD) or "progressive disease" (PD).

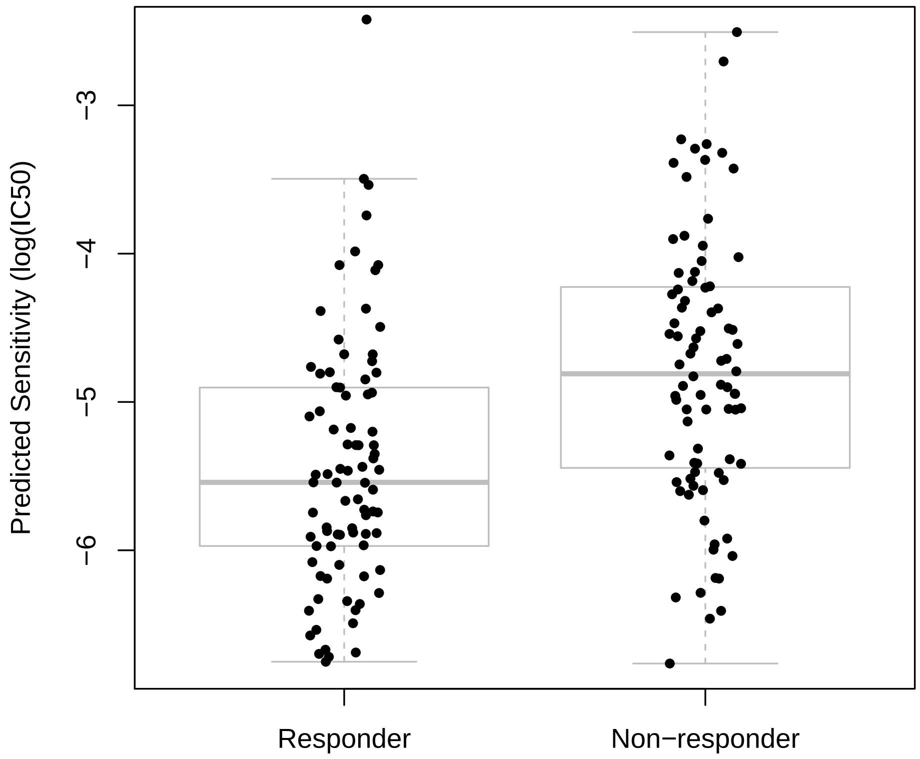

Figure 4: Stripchart and boxplot of the difference in predicted drug sensitivity between *in vivo* responders and non-responders to bortezomib, whose gene expression levels were measured using the U133B array. The predicted drug sensitivity is clearly lower in the "responder" group.

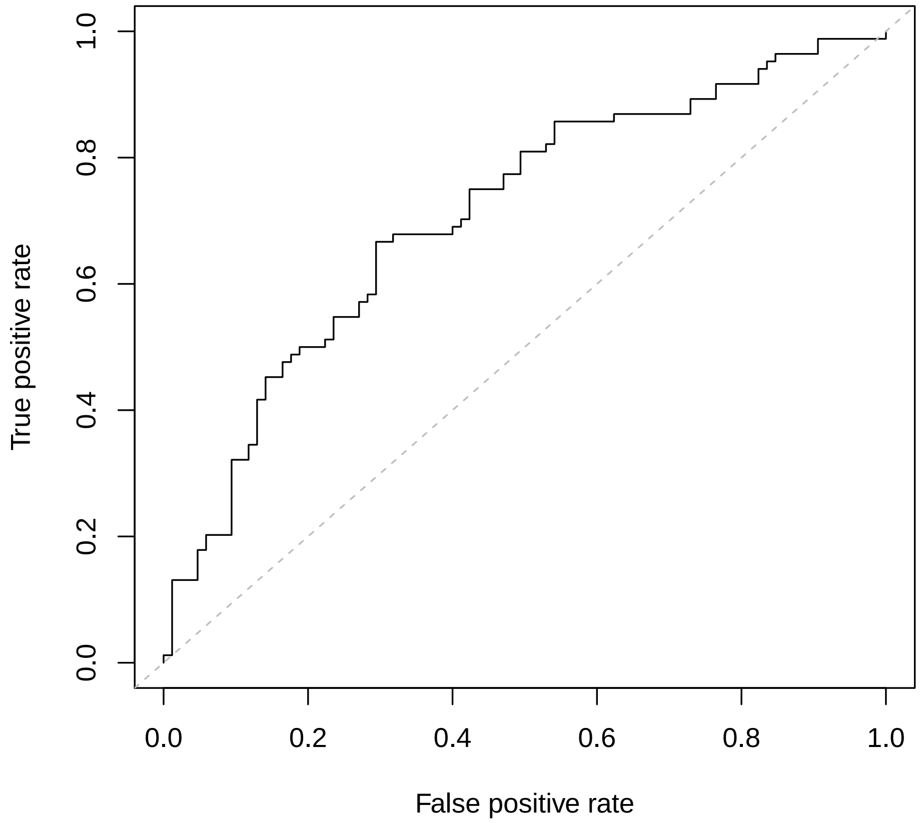

Figure 5: A ROC curve showing the proportion of true positives against the proportion of false positives as the classification threshold is varied, for predicted drug sensitivity values in the bortezomib clinical trial (individuals whose gene expression levels were measured using the U133B array).

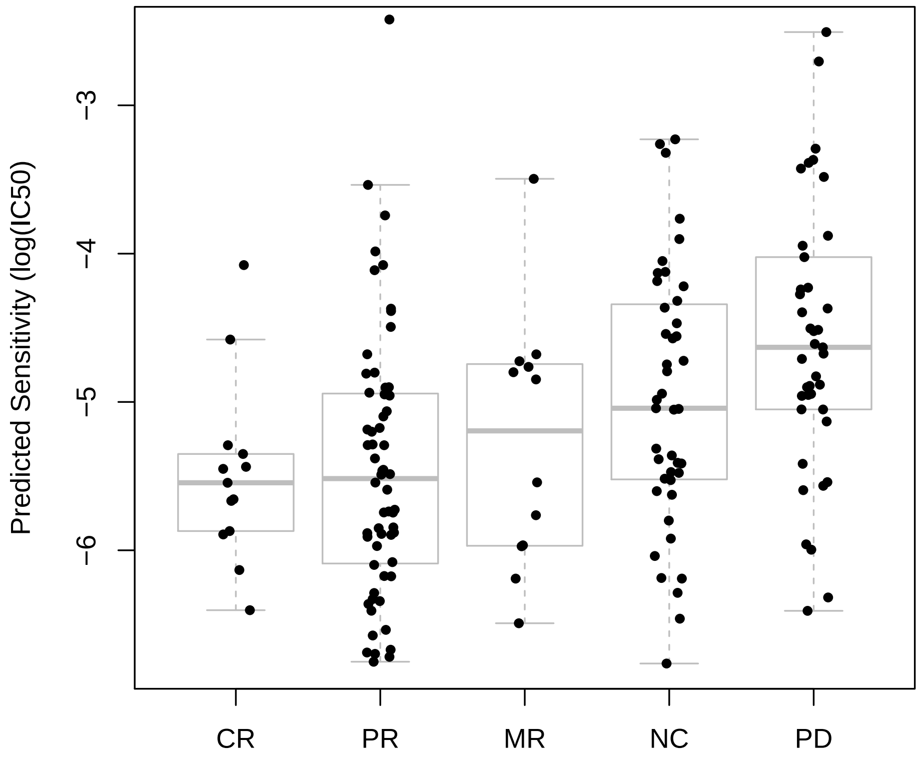

Figure 6: Stripchart and boxplot of the difference in predicted drug sensitivity in complete response (CR), partial response (PR), minimal response (MR), no change (NC) and progressive disease (PD) groups for individuals treated with bortezomib, whose gene expression levels were measured using the U133B array.

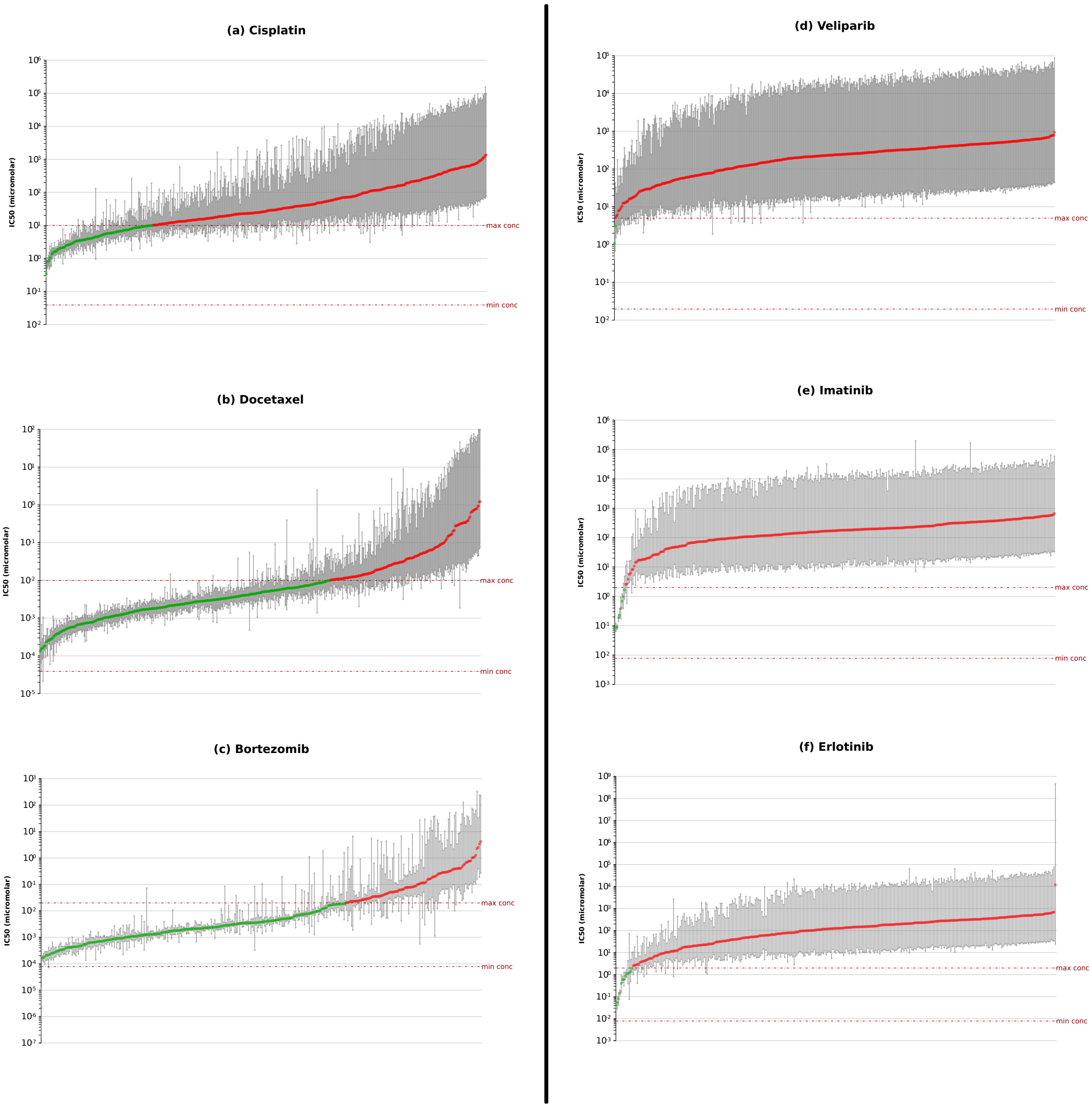

Figure 7: A plot showing drug sensitivity in each of the GDSC cell lines for (a) cisplatin, (b) docetaxel, (c) bortezomib, (d) veliparib, (e) imatinib, (f) erlotinib. In each plot cell lines are ordered by increasing IC50 (x-axis), the horizontal dashed red lines indicate the maximum and minimum drug screening concentration. All IC50 values outside this interval are estimated using extrapolated data (these points are highlighted in red) and thus have very large associated confidence intervals (shown as vertical gray lines). For all cytotoxic agents (left column; figs. a, b and c), many IC50s are reliably measured. For targeted agents (right column; figs. d, e and f), the vast majority cell lines are outside the screening window and thus IC50 values are estimated using extrapolated data and have very large confidence intervals (i.e. these IC50 values are "noisy"). Thus linear ridge regression should not be used to model this data and logistic regression must be used instead. These plots were downloaded from www.cancerrxgene.org (accessed August 2013).

Supplementary Tables

	Ridge Regression	Lasso	ElasticNet
Docetaxel	4.0×10^{-3}	0.01	0.01
Bortezomib	8.9×10^{-4}	0.06	1.3×10^{-3}
Erlotinib	5.3×10^{-4}	0.02	3.6×10^{-3}

Table 1: This table shows the p-values achieved for each dataset using Ridge, Lasso and ElasticNet regression. For each drug, p-values were calculated as described in the main text. Results for bortezomib were calculated using the Affymetrix U-133A arrays. All erlotinib results were derived from a logistic model fit using the 15 most sensitive and 55 most resistant cell lines (as described in the main text). The regularization parameters for each algorithm were selected as described in Methods.
